# A Novel Treatment Concept for Advanced Stage Mandibular Osteoradionecrosis Combining Isodose Curve Visualization and Nerve Preservation: A Prospective Pilot Study

**DOI:** 10.3389/fonc.2021.630123

**Published:** 2021-02-22

**Authors:** Gustaaf J. C. van Baar, Lars Leeuwrik, Johannes N. Lodders, Niels P. T. J. Liberton, K. Hakki Karagozoglu, Tymour Forouzanfar, Frank K. J. Leusink

**Affiliations:** ^1^Amsterdam UMC and Academic Centre for Dentistry Amsterdam (ACTA), Vrije Universiteit Amsterdam, Department of Oral and Maxillofacial Surgery/Pathology, Amsterdam, Netherlands; ^2^Amsterdam UMC, Vrije Universiteit Amsterdam, Medical Technology, 3D Innovation Lab, Amsterdam, Netherlands

**Keywords:** osteoradionecrosis, mandibular reconstruction, inferior alveolar nerve, treatment, computer-assisted surgery

## Abstract

**Background:**

Osteoradionecrosis (ORN) of the mandible is a severe complication of radiation therapy in head and neck cancer patients. Treatment of advanced stage mandibular osteoradionecrosis may consist of segmental resection and osseous reconstruction, often sacrificing the inferior alveolar nerve (IAN). New computer-assisted surgery (CAS) techniques can be used for guided IAN preservation and 3D radiotherapy isodose curve visualization for patient specific mandibular resection margins. This study introduces a novel treatment concept combining these CAS techniques for treatment of advanced stage ORN.

**Methods:**

Our advanced stage ORN treatment concept includes consecutively: 1) determination of the mandibular resection margins using a 3D 50 Gy isodose curve visualization, 2) segmental mandibular resection with preservation of the IAN with a two-step cutting guide, and 3) 3D planned mandibular reconstruction using a hand-bent patient specific reconstruction plate. Postoperative accuracy of the mandibular reconstruction was evaluated using a guideline. Objective and subjective IAN sensory function was tested for a period of 12 months postoperatively.

**Results:**

Five patients with advanced stage ORN were treated with our ORN treatment concept using the fibula free flap. A total of seven IANs were salvaged in two men and three women. No complications occurred and all reconstructions healed properly. Neither non-union nor recurrence of ORN was observed. Sensory function of all IANs recovered after resection up to 100 percent, including the patients with a pathologic fracture due to ORN. The accuracy evaluation showed angle deviations limited to 3.78 degrees. Two deviations of 6.42° and 7.47° were found. After an average of 11,6 months all patients received dental implants to complete oral rehabilitation.

**Conclusions:**

Our novel ORN treatment concept shows promising results for implementation of 3D radiotherapy isodose curve visualization and IAN preservation. Sensory function of all IANs recovered after segmental mandibular resection.

## Introduction

Osteoradionecrosis (ORN) of the jaws is a common side effect of radiation therapy (RT) (1–4). ORN is defined as the process where irradiated bone becomes necrotic and exposed for a time period of at least 3 months, and fails to heal (5–8). It affects the mandible, in particular the body, more often than the maxilla or any other bone of the head and neck area (9) and has an incidence in the mandible between 2% and 22% (10, 11). Although ORN is often diagnosed within 2 years after RT, there is a lifelong risk for this severe complication (12).

Risk factors associated with ORN are well documented (8, 13–15), with the most prominent being the radiation dose. A radiation dose more than 60 Gy is reported as high risk and 50–60 Gy as intermediate risk (4, 8, 15–17). In the management of ORN prevention is crucial since the process is irreversible and progression is difficult to control. Once ORN is diagnosed conservative measurements are indicated (18–20). For advanced stages of ORN these conservative measurements alone are not sufficient.

There are different ORN classification systems described in the literature, however the Notani classification (21) seems to be the most reliable for determining progression of ORN in the mandible (**Table 1**) (22). In advanced stage ORN (Notani stage III), segmental mandibular resection may be indicated (13, 18, 19). However, determining resection margins may be difficult as the extent, severity, and location of ORN do not always correlate with radiographical imaging (23).

**Table 1:** The Notani classification for mandibular osteoradionecrosis.

Stage I	Osteoradionecrosis confined to the alveolar bone
Stage II	Osteoradionecrosis limited to the alveolar bone or the mandible, or both above the mandibular alveolar canal
Stage III	Osteoradionecrosis that extended to the mandible under the level of the mandibular alveolar canal and osteoradionecrosis with a skin fistula or a pathological fracture, or both

Computer-assisted surgery (CAS) is well known in mandibular resection and reconstruction since its introduction by Hirsch in 2009 (24, 25) introducing high accuracy results and shortened operation time (26–30). In addition, CAS can facilitate incorporation of intensity modulated radiation therapy (IMRT) data in the virtual planning of segmental mandibular resection and reconstruction. As radiation dose seems to correlate with the risk for ORN (15), Kraeima et al. (2018) incorporated RT isodose curves in the virtual planning of the resection using a three-dimensional image of the administered RT dose of 50 Gy (31). With this patient-specific technique, the mandibular resection can be planned highly accurate out of the irradiated bone, leading to a minimally invasive mandibular resection.

Although the inferior alveolar nerve (IAN) is not directly affected in ORN cases, the nerve is often sacrificed during mandibular resection. Injury of the IAN may have a significant negative impact on quality of life as it may cause chronic pain (32). Additionally, maintaining sensorimotoric function of the lower lip and chin may be beneficial for oral function such as speech and mastication (33). Free handed preservation of the IAN is time consuming (34, 35) and includes a considerable risk of iatrogenic nerve injury (36). The use of CAS techniques for preserving the IAN during segmental mandibular resection has been evaluated by previous studies (37–40), showing promising results to prevent sensory disturbance of the lip and chin region. After segmental resection of necrotic bone, mandibular continuity can be restored with a vascularized bone flap covering the defect with non-irradiated soft-tissue (41). Currently, the fibula free flap (FFF) is the most commonly used reconstruction approach (42–44).

In this prospective pilot study we combined RT isodose curve visualization with 3D guided IAN preservation in order to improve quality of life. Research to date has not yet combined these two CAS techniques. The accuracy of the mandibular reconstructions were evaluated postoperatively (45). In all cases the postoperative IAN sensory function was objectified and compared with the preoperative function. A visual analog scale-based questionnaire was used to evaluate subjective sensibility.

## Materials and Methods

### Patients

This study was conducted in the Department of Oral and Maxillofacial Surgery/Oral Pathology, Amsterdam UMC, VU University Medical Center Amsterdam (the Netherlands) and was approved by the Medical Ethics Review Committee of VU University Medical Center (FWA 00017598). Between November 2017 and March 2019 all ORN stage III patients (minimum age of 18 years) who received IMRT in the past with an indication for segmental mandibular resection were included. Patients with diagnosed malignancies were excluded.

### Preoperative Imaging

A preoperative multi-detector row computed tomography (MDCT) scan (kVp 120, mAs 300, slice thickness 0.625 mm) was made of the skull using a GE Discovery CT750 HD 64-slice MDCT scanner (GE Healthcare, Little Chalfont, Buckinghamshire, UK). The lower leg was scanned with CT angiography (CTA) for visualization of the fibula including vessel anatomy. Both Digital

Imaging and Communications in Medicine (DICOM) files were uploaded in Mimics Medical 21.0 software (Materialise, Leuven, Belgium) and converted into 3D models using the thresholding tool; voxels with an HU above a selected threshold value are included in the ROI and transformed into 3D surface models in the Standard Tessellation Language (STL) file format (46).

### Isodose Curve Visualization

**Figure 1:**
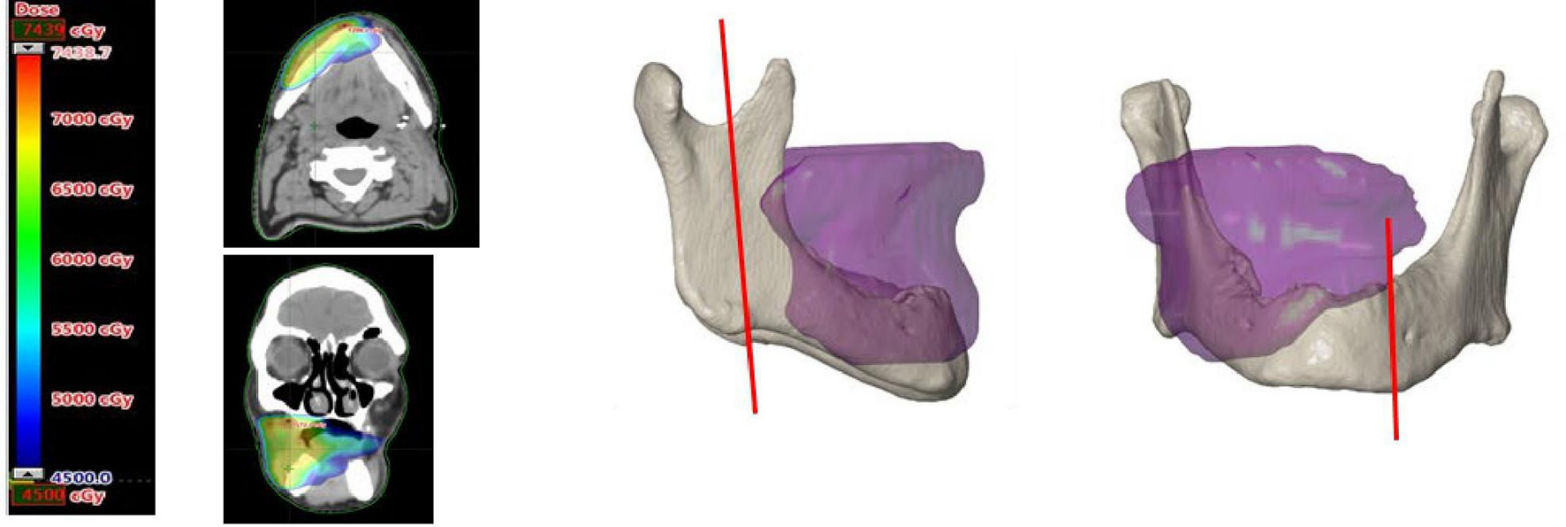
On the left an axial and coronal CT image with superimposed the field of view exposed to 50 Gy or more. On the right a 3D image of the superimposed 50 Gy field on the 3D model of the mandible. The vertical red lines on the mandible mark the planned resection margins.

In the RT software (Eclipse™, external beam planning V15.6, Varian medical systems, Palo Alto, CA, USA) the 50 Gy isodose borders from the IMRT data were determined, converted into a 3D model, and superimposed on the 3D model of the mandible. Subsequently the mandibular resection margins were determined on the mandible, taking into account the above mentioned isodose curve visualization and the optimal direction of the osteotomy planes for FFF reconstruction. Remnant mandibular bone outside the resection was checked for the signs of ORN on CT (mono- or bicortical destruction, central necrosis, and sequestration) (23). **Figure 1** shows an example of a 3D model with the bone that had been exposed to a high risk dose of 50 Gy or more.

### IAN Localization

**Figure 2:**
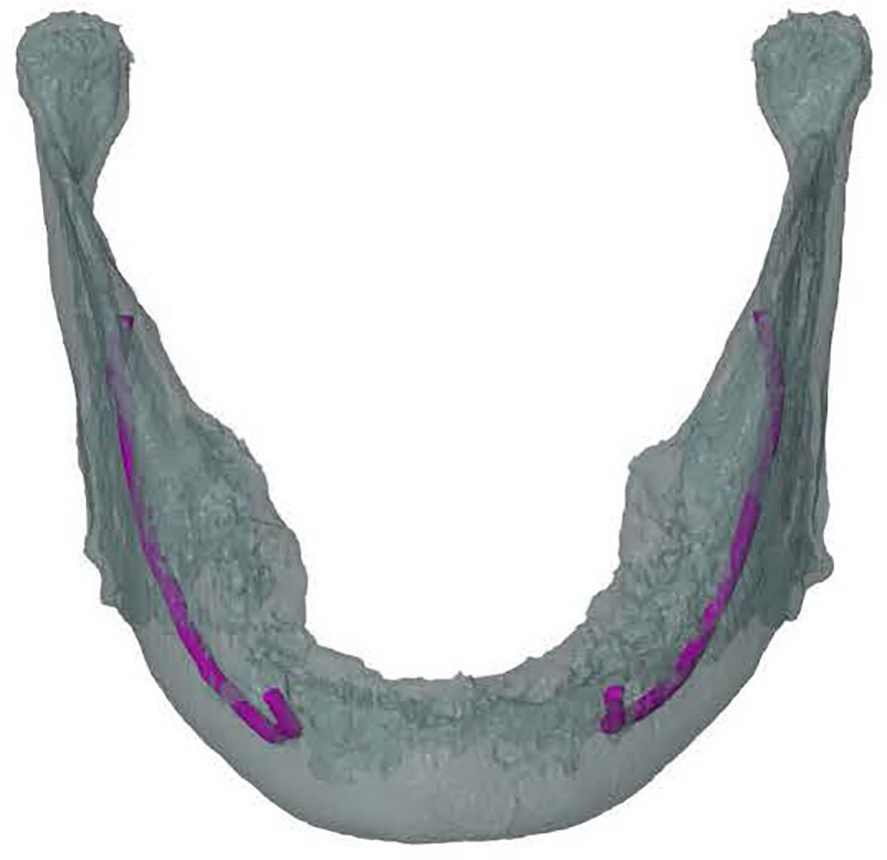
Frontal view of a 3D model of the mandible including inferior alveolar nerve tracing (in purple) on the left and the right.

The mandibular canal was traced using the tool “trace thin structure” in Mimics Medical 21.0 in a coronal view from the mandibular foramen to the mental foramen in steps of 2 mm. Once the canal was marked, the tracing was checked in sagittal view of the CT scan. The same procedure was used for the other side. The traced canals were exported in STL format. The mandible was also segmented and exported in STL format (**Figure 2**).

### Guide Design and Manufacturing

**Figure 3:**
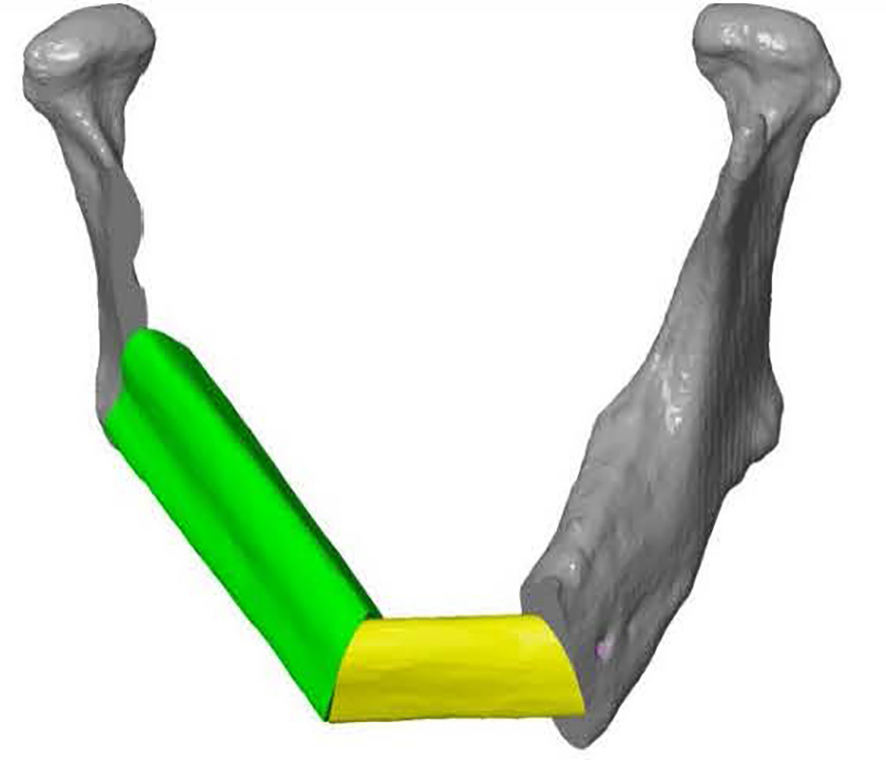
Frontal view of a 3D model of the virtual planned reconstruction in Proplan CMF.

**Figure 4:**
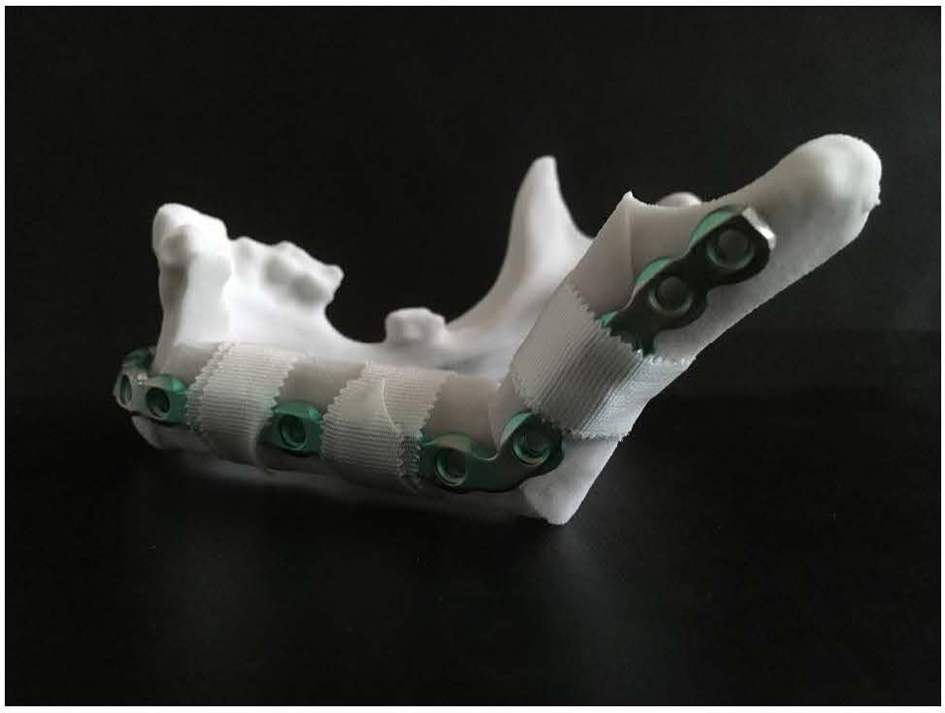
KLS Martin 2.7 mm reconstruction plate bent on a 3D model of the reconstructed mandible, creating a patient specific reconstruction plate.

ProPlan CMF 2.1. software (Materialise, Leuven, Belgium) was used to design the osteotomy planes on the mandible and to determine the optimal position and configuration of the fibula segments to reconstruct the mandibular defect (25) (**Figure 3**). This virtual model was 3D printed and figured as a template to pre-bent a KLS Martin 2.7 mm reconstruction plate into a patient specific reconstruction plate (PSRP) (**Figure 4**). Subsequently a CT scan of the PSRP was made, converted to STL format, and used further along in the virtual planning to determine the locations of the fixation screws.

**Figure 5:**
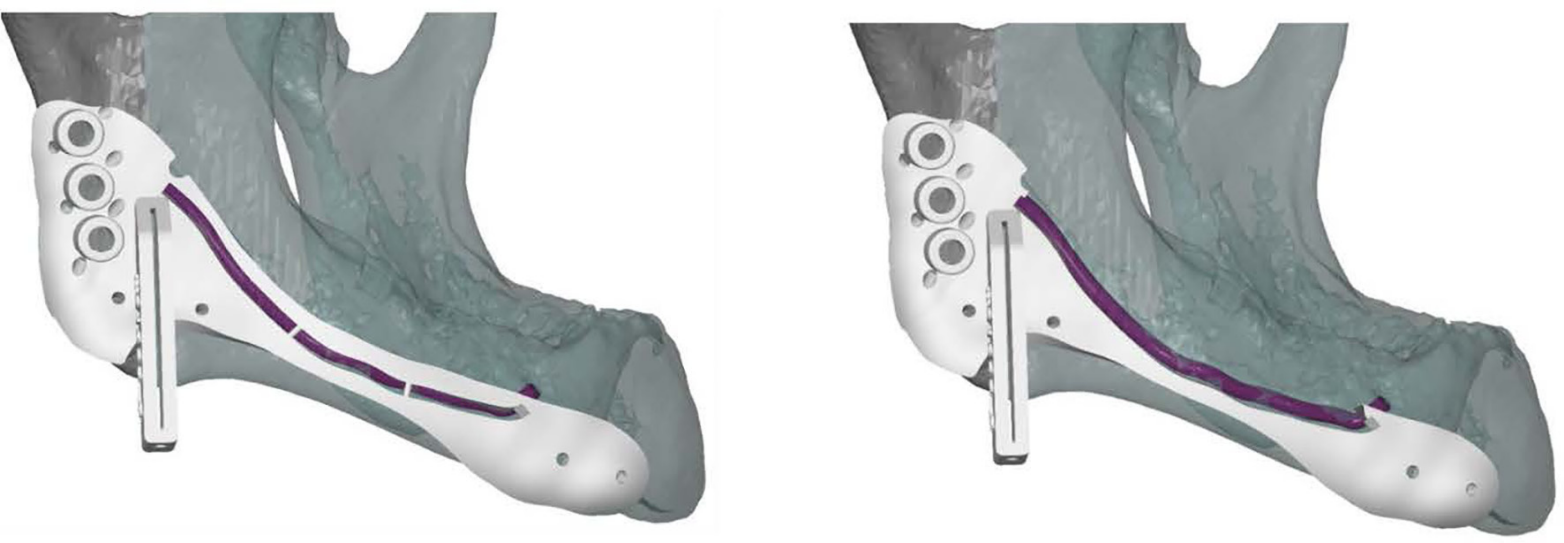
Lateral view of a cutting guide, allowing two-step exteriorization of the inferior alveolar nerve. The purple line indicates the inferior alveolar nerve. The guide shows three drilling holes on the left for plate fixation surrounded by small holes for water cooling during drilling. The guide also includes a saw box to create the osteotomy plane. The two small holes surrounding the saw box and the two small holes on the right side of the guide are used for fixation.

All cutting guides were designed using 3-Matic Medical software 14.0 (Materialise, Leuven, Belgium). To preserve the IAN a two-step mandibular guide was created with a free margin of 2 mm cranially to allow two-step deroofing of the superior and lateral cortex of the mandibular canal. When using CT data for manual mandibular canal tracing a safety zone of 1.7 mm should be taken into account (47). A template design with cutting guides is shown in **Figure 5**. The STL files of the cutting guides were 3D printed in PA12 material in compliance to ISO 13485 and sterilized.

### Surgical Procedure

**Figure 6:**
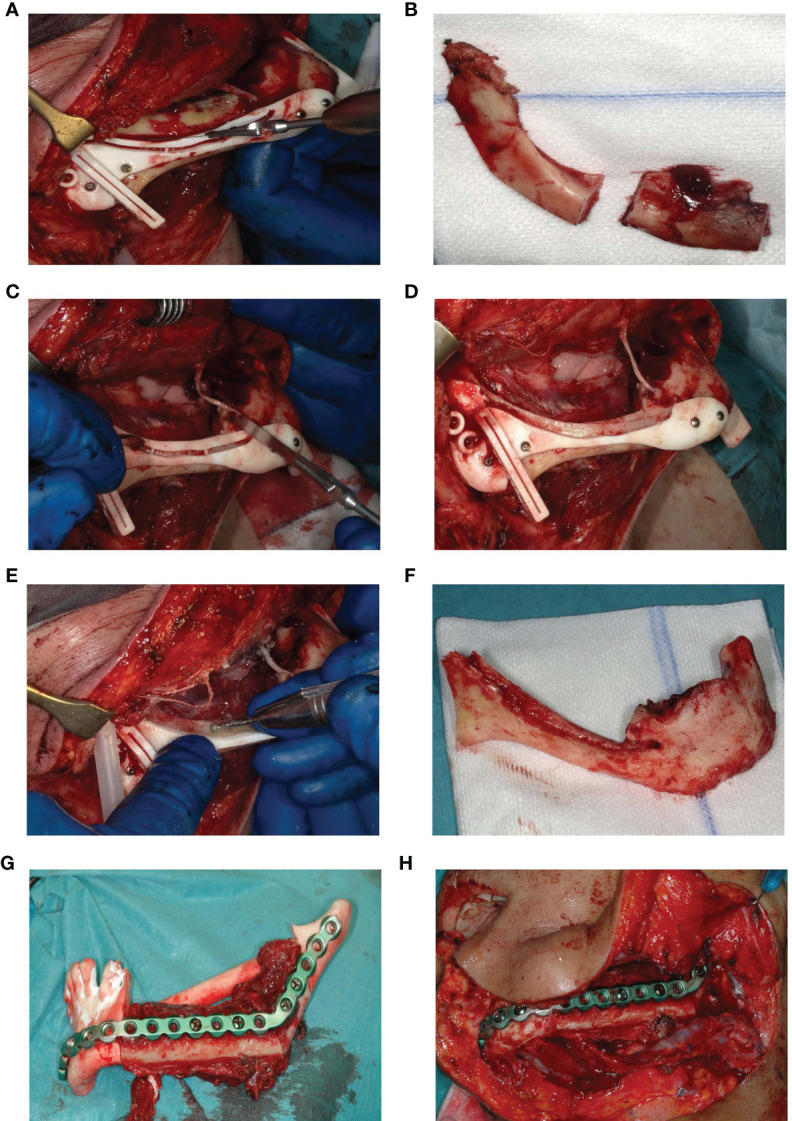
**(A)** Deroofing of the upper part of the mandible. **(B)** Removed upper part of the mandible after two vertical osteotomies on both sides. **(C)** Removal of the upper part of the guide. **(D)** Accessible buccal bone and 2 mm roof of the IAN. **(E)** Buccal corticotomy to expose the IAN. **(F)** Resected part of the mandible without the right IAN. **(G)** Fitting of the fibula segments fixated to the patient specific reconstruction plate into a 3D printed mandible including the planned resection. **(H)** Reconstruction *in situ*.

Once surgical access and mandibular exposure was obtained, the cutting guide was positioned and fixed to the mandible with four titanium screws (4× KLS Martin 1.5. × 7 mm screws). The resection started with a horizontal osteotomy 2 mm above the mandibular canal (**Figure 6A**) and completed with two vertical osteotomies on both sides. The superior part of the mandible was subsequently removed (**Figure 6B**). After removing the upper part of the IAN cutting guide with a reciprocal saw (**Figure 6C**), the superior and buccal cortex of the mandibular canal are exposed (**Figure 6D****)** and can be removed with a hard steel burr (**Figure 6E**). The IAN can be exposed along its entire path with this technique. Once secured, mandibular resection is proceeded as planned (**Figure 6F**). The full surgical process is shown in **Figure 6**.

### Outcome Evaluation

Accuracy of the mandibular reconstruction was evaluated according to the evaluation method for computer-assisted surgery in mandibular reconstruction described by Van Baar et al (45, 46)..

A review of Poort et al. (2009) recommends the use of Semmes Weinstein monofilaments as a reliable and reproducible test for measuring sensation in the mental nerve area, in combination with a patient’s subjective function by using the visual analogue scale (VAS). We used five Semmes-Weinstein monofilaments (Baseline^®^ tactile™ monofilament evaluator case) to objectify sensory function of the IAN (in order 300, 40.0, 2.0, 0.4, and 0.07 gram) (48). The monofilaments were placed perpendicular to the front of the chin and lower lip and pressed until the filament begins to deform. At this point, a known reproducible pressure is applied. The monofilaments are placed on a grid of 24 locations on the front of the chin and lower lip (i.e. innervation of one IAN was divided into a 12-point grid). Each approach at each individual measuring point of the grid contains two moments of attention in which either a test stimulus or a fake stimulus is applied. The fake stimulus is performed by approaching the lower lip/chin with an averted monofilament. The order of test/fake stimulus in the two moments of attention is randomized. A stimulus (test or fake) is preceded by the words spoken: “test 1” and “test 2.” After each offer, the patient indicates whether the test stimulus was administered during attention moment 1 or 2. If the patient does not know exactly, they have to guess (“two alternative forced choice” test procedure). The sensitivity test score is positive if the test stimulus is correctly detected in seven consecutive offers. With seven offers, the chance that a correct result will be achieved by means of guessing is less than 0.01 (<0.5). At the first error in the series of seven, the test can be terminated immediately with a negative result (49). The total amount of positive reactions were added up for all five monofilaments on each of the 12 locations (i.e. no function of the IAN resulted in a 12 × 0 score of 0 and full function resulted in a 12 × 5 score of 60). Eventually, the score was converted into a score between 0 and 5 for statistical analysis.

For subjective IAN sensory function two Visual Analog Scales (VAS) were used (**Supplementary Figure 1**), asking the following questions: “How would you describe the sensation of your lower lip and chin. Place a vertical mark on the line below to indicate the sensation on your lower lip and chin today” and “Place a vertical mark on the line below to indicate the level of sensation on your lower lip and chin you find acceptable in daily life.” Both vertical marks were transformed to a score on a scale from 1 “no feeling” to 10 “normal feeling.”

Subjective and objective function of the IAN were determined one day preoperatively (T0) and 2–4 weeks (T1), 2–3 months (T2), 6–7 months (T3), and 1 year or more (T4) postoperatively.

### Statistical Analysis

SPSS Software package (version 26.0 Inc., Chicago, IL, USA) was used for statistical analysis. A paired samples T-test was executed for both subjective and objective IAN function between T0 measurements and T1-T4 measurements. Statistical significance was reached with a two-tailed p value of <0.05. As T0 measurements were expected to be different for cases with a pathologic fracture and those without, these cases were analyzed separately.

## Results

### Patients

Between November 2017 and March 2019 five patients were included with a mean age of 53.4 years (49,50,51,52,53,54,55,56,57). A total of seven IAN were preserved (two patients required bilateral IAN preservation). All patient characteristics are shown in **Table 2**. No patients developed peri-operative complications, in particular there were no clinical or radiological signs of recurrent ORN or non-union for at least 1 year after surgery.

**Table 2:** An overview of the included patients and their characteristics.

Nr.	Age	Sex	Primary diagnosis	TNM	Treatment	Secondary diagnosis	Included IAN
1	49	F	Tonsil R SCC	T2N1	Chemoradiotherapy: 70 Gy	ORN + pathologic fracture	R
2	57	M	Floor of mouth R+L SCC	T3N1	Surgery, Radiotherapy: 70 Gy	ORN	L + R
3	54	F	Tonsil L SCC	T1N2a	Radiotherapy: 70 Gy	ORN + pathologic fracture	L
4	56	M	Buccal mucosa R SCC	T1N0	Surgery Radiotherapy: 66 Gy	ORN	R
			Buccal mucosa L SCC	T2N0	Surgery		
5	51	F	Floor of mouth L SCC	T2N2	Surgery, Chemoradiotherapy: 55 Gy	ORN	L + R

F, female; M, male; R, right; L, left; SCC, squamous cell carcinoma; ORN, Osteoradionecrosis.

### Accuracy

**Figure 7:**
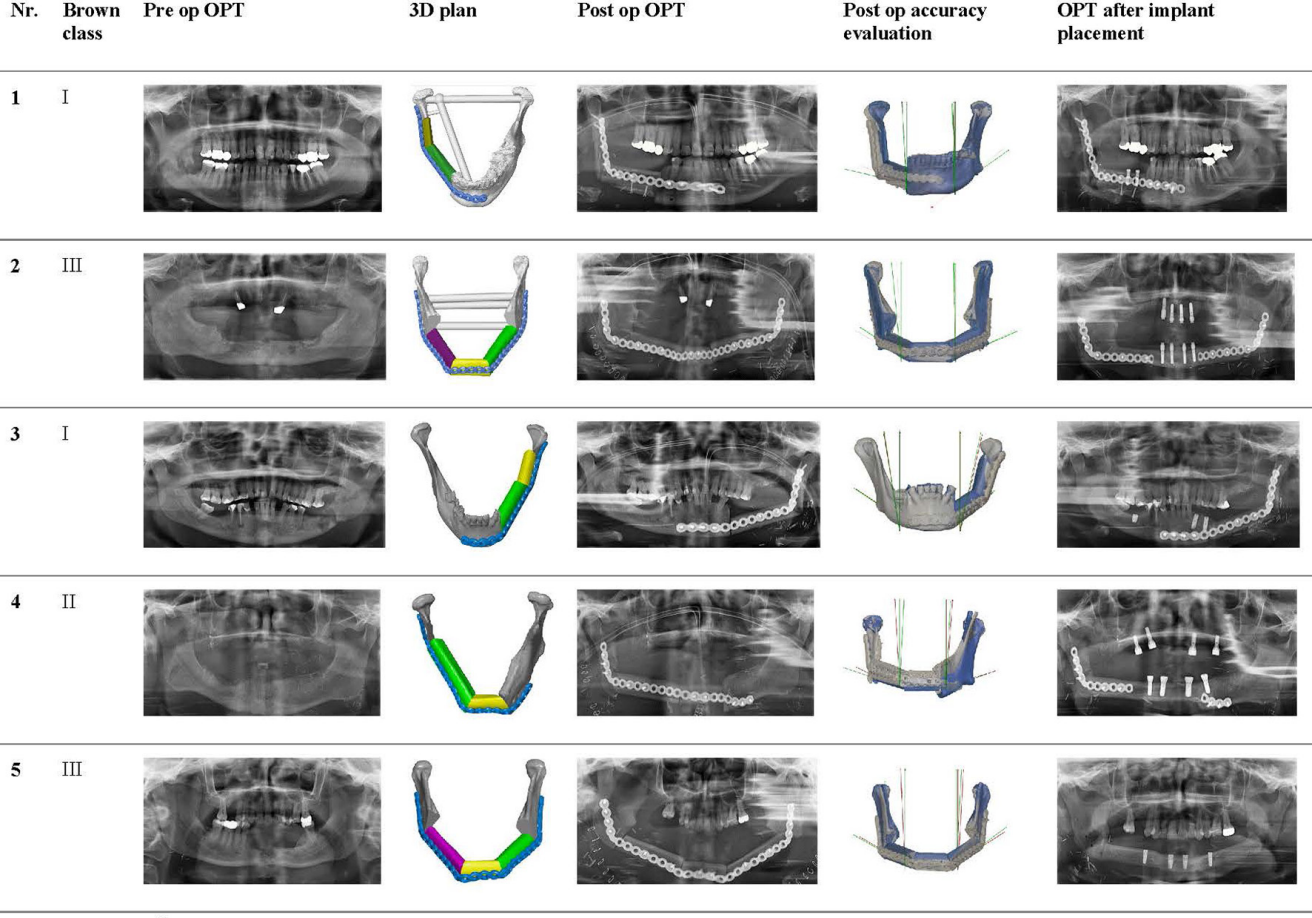
Panoramic radiographs of all included cases preoperatively, postoperatively, and after implant placement, with the 3D plan and accuracy measurements added in between.

**Table 3** shows all angle deviations (AD) in degrees per angle. The mandibular defect classification of Brown et al. was used (50). **Figure 7** shows the panoramic radiographs preoperatively, postoperatively, and after implant placement, with the 3D plan and accuracy measurements added in between.

**Table 3:** Angle deviations in degrees (°) between the preoperative virtual plan and the postoperative result.

Nr.	Brown class	Axial		Coronal		Sagittal	
		L	R	L	R	L	R
**1**	I	2.40	6.42	0.54	0.06	0.28	0.10
**2**	III	0.17	2.27	0.77	2.99	1.93	1.10
**3**	I	0.94	0.12	0.73	0.49	2.17	0.11
**4**	II	2.45	2.84	1.03	0.32	3.78	1.49
**5**	III	0.23	1.30	0.10	2.39	7.47	2.78

### Nerve Evaluation

**Figure 8:**
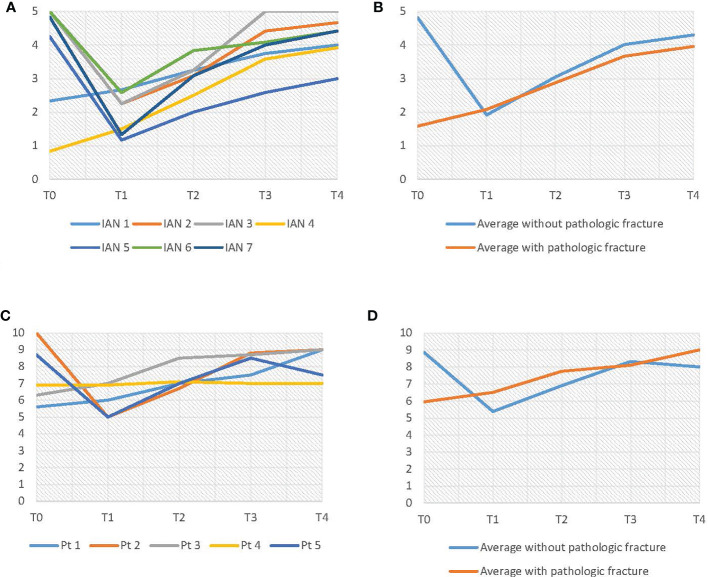
**(A)** Results of the light touch test. **(B)** Average results of the light touch test, differentiated on pathologic fracture. **(C)** VAS-score results. **(D)** Average VAS-score results, differentiated on pathologic fracture.

The objective IAN function results are shown in **Figures 8A, B**. As can be seen in **Figure 8B**, there were two patients with a pathologic fracture (IAN 1 and IAN 4). Patients without a pathologic fracture had an average preoperative score of 4.8. At T1 these patients had an average score of 1.9, which was significantly lower than T0 (p = 0.00) (**Table 4**). However, the objective IAN function improved at T2 up to an average score of 4.3 at T4 for patients without a pathologic fracture (p = 0.07) (**Table 5**).

**Table 4:** Paired samples T-test between T0 and T1 for objective IAN function in patients without a pathologic fracture (n = 5). α = 0,05.

	Mean (SD)	Mean difference (SD)	t-value	p-value
**T0**	4,8167 (0,32489)	2,9 (0,40995)	15,818	0,000
**T1**	1,9167 (0,62639)			

**Table 5:** Paired samples t-test T0–T4. Light touch test. Without pathologic fracture (n = 5). α = 0,05.

	Mean (SD)	Mean difference (SD)	t-value	p-value
**T0**	4,8167 (0,32489)	0,51667 (0,46173)	2,502	0,067
**T4**	4,3 (0,76513)			

As shown in **Figure 8C, D**, the subjective IAN function (VAS-scores) showed similar results as the objective IAN function (**Figures 8C, D**). The light touch test results for the three control IANs were consistent throughout every evaluation moment.

## Discussion

ORN of the jaw is still a common side effect of RT, even after the introduction of intensity-modulated radiotherapy (1–3). ORN can be treated with conservative measures, but in more severe cases (Notani stage III) a segmental resection followed by a vascularized reconstruction flap should be considered (13, 18, 19). Due to new CAS techniques, preservation of the IAN during mandibular resection is more feasible than ever. Previous studies have evaluated these new CAS techniques for preservation of the IAN during mandibular resection (37–40). These studies have published promising results for postoperative sensory disturbance, but none of them used reliable and reproducible clinical neurosensory tests which are advised for sensory evaluation of trigeminal nerve branches (48, 51). In addition, the study groups consisted of patients with different preoperative diagnoses.

This study included only patients diagnosed with ORN. The sensory disturbance of the IAN was evaluated using the light touch test with Semmes-Weinstein monofilaments. We also used a VAS-score questionnaire to measure subjective feeling. Poort et al. recommended to use a follow-up regimen of 1 week, 1 month, 3 months, and 1 year (48). However, in this study we did not follow this exact regimen for patient load reasons.

Our results show that IAN preservation using CAS is possible. None of the patients experienced reoccurring ORN within at least 1 year, which suggests enough infected bone was resected. This study suggests that the use of RT isodose curves set to 50Gy can therefore be safely implemented in determining osteotomy planes. Our results show that there was some sensory disturbance of the IAN after surgery, but the mental nerve area regains its sensitivity each following evaluation moment to almost its preoperative sensitivity after 1 year. The cases with a pathological fracture, which already had an IAN sensitivity disturbance, regained even more sensitivity than before the surgery.

The statistical analysis of the average IAN sensitivity (light touch test and VAS) of the “pathological fracture” cases (n = 2) did not show a significant difference, this may be a result of the low case number. The “no pathological fracture” cases did show statistical significant results during analysis. We did not take the double inclusion of patients (two “bilateral patients”) into account, which may be a weakness of the executed analysis. The three unaffected IANs, used as controls, did not show different sensitivity levels for each evaluation moment, meaning the light touch test with Semmes-Weinstein monofilaments was consistent.

The sensitivity survival and recovery indicates that the nerve tracking technique was sufficient: mandibular canal tracing in steps of 2 mm in coronal view on the CT of the skull. For the design of the IAN preservation guide we considered the discrepancy of the virtual traced IAN location and the actual location by designing a two-step deroofing process. Once the upper part of the guide was removed, the IAN was still covered with bone and could be carefully exteriorized. By approaching the IAN from the buccal side (buccal corticotomy), it could be lifted easily from the mandibular canal.

In our treatment concept, a virtual model was 3D printed and figured as a template to pre-bent a reconstruction plate into a patient specific reconstruction plate (PSRP) (**Figure 4**). Subsequently a CT scan of the pre-bent PSRP was made, converted to STL format, and used further along in the virtual planning. This has the same advantages as a 3D printed titanium patient specific reconstruction plate, but saves on the high costs of the selective laser sintering manufacturing technique (52). Another advantage of this treatment concept is that no third party is involved in the planning phase, which speeds up the workflow for hospitals with its own 3D lab. Our systematic review of accuracy in mandibular reconstruction using CAS showed that 14 out of the 42 included studies used a standard reconstruction plate which was pre-bent on a 3D printed model of the virtually planned reconstruction and 12 studies used a 3D printed PSRP. Even though the studies were difficult to compare, there were no striking differences in accuracy or postoperative complications between the studies using a pre-bent reconstruction plate or a 3D printed PSRP (53).

The measured accuracy of the reconstructions did not show any extreme deviations. Since the accuracy is on such a high level, we believe it is possible to perform computer guided mandibular reconstructions with direct dental implant placement in ORN cases. Especially since in ORN cases the neomandible is constructed with well vascularized donor bone and postoperative RT is not indicated. All patients received dental implants after an average time of 11.6 months (min. 8/max. 19 months). The use of immediately placed dental implants will improve dental rehabilitation time significantly. Any data on acceptable outcome ranges regarding immediately placed dental implants during mandibular reconstruction has yet to be published.

A shortcoming of this study was the low case number, caused by small numbers of ORN cases. Future multi-center prospective studies need to be carried out in order to validate the results of our novel treatment concept.

## Conclusion

Our novel ORN treatment concept shows promising results for implementation of 3D radiotherapy isodose curve visualization and IAN preservation. Sensory function of all IAN recovered after segmental mandibular resection.

## Data Availability Statement

The original contributions presented in the study are included in the article/**Supplementary Material**. Further inquiries can be directed to the corresponding author.

## Ethics Statement

The studies involving human participants were reviewed and approved by the Medical Ethics Review Committee of VU University Medical Center (FWA 00017598). The ethics committee waived the requirement of written informed consent for participation.

## Author Contributions

Conceived and designed the analysis: GV, JL, LL, TF, and FL. Collected the data: GV, LL, NL, KK, JL, TF, and FL. Contributed data or analysis tools: GV, LL, JL, and KK. Performed the analysis: GV, LL, and JL. Wrote the paper: GV, LL, JL, and FL. All authors contributed to the article and approved the submitted version.

## Conflict of Interest

The authors declare that the research was conducted in the absence of any commercial or financial relationships that could be construed as a potential conflict of interest.

## Supplementary Material

Supplementary Figure 1 Two Visual Analogue Scales (VAS) were asked to the patient to evaluate subjective sensory function of the inferior alveolar nerve.
