# Supplementary figures and images for: A Novel Treatment Concept for Advanced Stage Mandibular Osteoradionecrosis Combining Isodose Curve Visualization and Nerve Preservation: A Prospective Pilot Study
Source: Front Oncol. 2021 Feb 22;11:630123. doi: 10.3389/fonc.2021.630123 (PMC7937888; doi:10.3389/fonc.2021.630123)

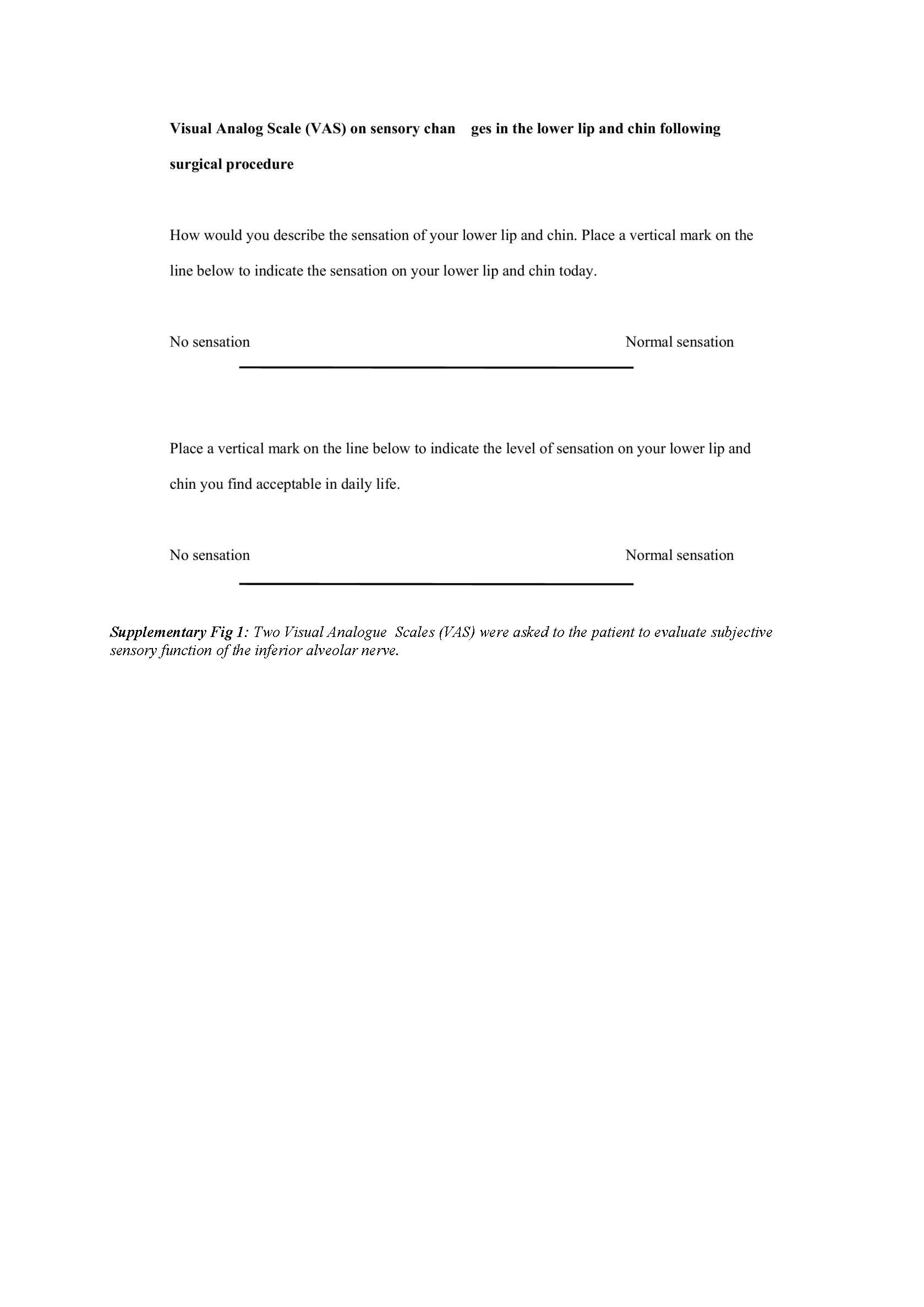

Supplement: Supplementary Figure 1 — Two Visual Analogue Scales (VAS) were asked to the patient to evaluate subjective sensory function of the inferior alveolar nerve. [file Image_1.jpeg]
